# An “All-Data-on-Hand” Deep Learning Model to Predict Hospitalization for Diabetic Ketoacidosis in Youth With Type 1 Diabetes: Development and Validation Study

**DOI:** 10.2196/47592

**Published:** 2023-07-18

**Authors:** David D Williams, Diana Ferro, Colin Mullaney, Lydia Skrabonja, Mitchell S Barnes, Susana R Patton, Brent Lockee, Erin M Tallon, Craig A Vandervelden, Cintya Schweisberger, Sanjeev Mehta, Ryan McDonough, Marcus Lind, Leonard D'Avolio, Mark A Clements

**Affiliations:** 1 Health Services and Outcomes Research Children's Mercy - Kansas City Kansas City, MO United States; 2 Department of Endocrinology Children's Mercy - Kansas City Kansas City, MO United States; 3 IRCCS Bambino Gesù Children's Hospital Rome Italy; 4 Cyft, Inc Cambridge, MA United States; 5 Center for Healthcare Delivery Science Nemours Children's Health Jacksonville, FL United States; 6 Joslin Diabetes Center Boston, MA United States; 7 Department of Medicine NU-Hospital Group Uddevalla Sweden; 8 Department of Molecular and Clinical Medicine University of Gothenburg Gothenburg Sweden; 9 Department of Internal Medicine Sahlgrenska University Hospital Gothenburg Sweden

**Keywords:** type 1 diabetes, T1D, diabetic ketoacidosis, DKA, machine learning, deep learning, artificial intelligence, AI, recurrent neural network, RNN, long short-term memory, LSTM, natural language processing, NLP

## Abstract

**Background:**

Although prior research has identified multiple risk factors for diabetic ketoacidosis (DKA), clinicians continue to lack clinic-ready models to predict dangerous and costly episodes of DKA. We asked whether we could apply deep learning, specifically the use of a long short-term memory (LSTM) model, to accurately predict the 180-day risk of DKA-related hospitalization for youth with type 1 diabetes (T1D).

**Objective:**

We aimed to describe the development of an LSTM model to predict the 180-day risk of DKA-related hospitalization for youth with T1D.

**Methods:**

We used 17 consecutive calendar quarters of clinical data (January 10, 2016, to March 18, 2020) for 1745 youths aged 8 to 18 years with T1D from a pediatric diabetes clinic network in the Midwestern United States. The input data included demographics, discrete clinical observations (laboratory results, vital signs, anthropometric measures, diagnosis, and procedure codes), medications, visit counts by type of encounter, number of historic DKA episodes, number of days since last DKA admission, patient-reported outcomes (answers to clinic intake questions), and data features derived from diabetes- and nondiabetes-related clinical notes via natural language processing. We trained the model using input data from quarters 1 to 7 (n=1377), validated it using input from quarters 3 to 9 in a partial out-of-sample (OOS-P; n=1505) cohort, and further validated it in a full out-of-sample (OOS-F; n=354) cohort with input from quarters 10 to 15.

**Results:**

DKA admissions occurred at a rate of 5% per 180-days in both out-of-sample cohorts. In the OOS-P and OOS-F cohorts, the median age was 13.7 (IQR 11.3-15.8) years and 13.1 (IQR 10.7-15.5) years; median glycated hemoglobin levels at enrollment were 8.6% (IQR 7.6%-9.8%) and 8.1% (IQR 6.9%-9.5%); recall was 33% (26/80) and 50% (9/18) for the top-ranked 5% of youth with T1D; and 14.15% (213/1505) and 12.7% (45/354) had prior DKA admissions (after the T1D diagnosis), respectively. For lists rank ordered by the probability of hospitalization, precision increased from 33% to 56% to 100% for positions 1 to 80, 1 to 25, and 1 to 10 in the OOS-P cohort and from 50% to 60% to 80% for positions 1 to 18, 1 to 10, and 1 to 5 in the OOS-F cohort, respectively.

**Conclusions:**

The proposed LSTM model for predicting 180-day DKA-related hospitalization was valid in this sample. Future research should evaluate model validity in multiple populations and settings to account for health inequities that may be present in different segments of the population (eg, racially or socioeconomically diverse cohorts). Rank ordering youth by probability of DKA-related hospitalization will allow clinics to identify the most at-risk youth. The clinical implication of this is that clinics may then create and evaluate novel preventive interventions based on available resources.

## Introduction

### Background

Despite advances in technologies and insulin analogs used to treat type 1 diabetes (T1D), 7% to 10% of youth and young adults with preexisting T1D in the United States still experience preventable hospital admissions for diabetic ketoacidosis (DKA) annually; this rate is increasing [1-3]. DKA is a severe metabolic decompensation caused by absolute insulin deficiency. DKA is also a leading cause of morbidity and mortality in youth with T1D, accounting for approximately 50% of all deaths in this population. Episodes can lead to dangerous complications such as long-term neurocognitive impairment, cerebral edema, coma, or even death [4-6]. In 2014, the mean hospital charge was US $26,566 per DKA admission, with the aggregate US national charges for DKA being US $5.1 billion [3]. Most studies pertaining to DKA risk prediction in youth have relied on a limited number of discrete variables available in the electronic health record (EHR) and on conventional statistical models, such as logistic regression, which do not consider changes in predictors or recurrence of discrete events over time [7-9].

Prior research has applied machine learning and deep learning to EHR data to forecast health outcomes but not yet to predict DKA among children with T1D [10]. The ability to accurately predict and effectively intervene to prevent hospital admissions for DKA would support the achievement of the quadruple aim of improving population health, reducing the cost of care, improving patient experience, and improving the work-life balance of health providers [11]. Many clinics providing care for individuals with T1D seek to improve the quality of health of their clinic populations by using population data housed in EHRs, enterprise data warehouses, or data repositories governed by learning health networks. Forecasting with such data may allow for earlier intervention before an adverse health outcome occurs [12].

### Objective

We constructed a predictive model using a recurrent neural network–based approach suited to processing time series and other sequential data [13]. We specifically developed and evaluated the performance characteristics of a long short-term memory (LSTM) model to predict the 180-day risk of DKA-related hospitalization among youth with T1D [14].

## Methods

### Study Design

We developed a model to predict DKA-related hospitalizations within the T1D population of diabetes centers. We considered youth with the appropriate International Classification of Diseases, Ninth and Tenth Revisions, codes to have T1D. Autoantibody and C-peptide laboratory results and expert chart review were used to confirm the diagnosis. We chose to develop the model for youth aged 8 to 18 years, as this age range represents most of the hospital admissions for DKA at the institution.

### Source Data

The source data were derived from the Cerner Millennium Electronic Medical Record System. Data used for model development and validation included demographic data, discrete clinical observations (laboratory results, vital signs, anthropometric measures, diagnosis, and procedure codes), medications, visit counts by type of encounter, number of historic DKA episodes, number of days since last DKA admission, patient-reported outcomes (answers to clinic intake questions), and data features derived from diabetes- and nondiabetes-related clinical notes via natural language processing (NLP). Demographic data included sex (female or male), age (in years), ethnicity (non-Hispanic or Hispanic), race (Asian, Black or African American, White, other, or unknown), and insurance type (public=Medicaid, other, government, or competitive medical plan and private=commercial, Blue Cross, or self-pay). For periods leading up to the prediction period, the counts of each clinical note type and the total words for each note type for the 20 most common note types were recorded. The counts for the 100 most common words and the 100 most common 2-word phrases were recorded as data features.

### Feature Generation for the LSTM Model

The handling of data features varied by feature type. Structured clinical data, comprising Current Procedural Terminology codes, diagnosis codes (International Classification of Diseases, Ninth and Tenth Revisions), and Systematized Nomenclature of Medicine Clinical Terms codes, were included in the model development. When an individual had multiple encounters on the same day, the corresponding Concept Unique Identifier (CUI) codes were grouped together, with each CUI code recorded only once. The counts per period for the 200 most common CUI codes were recorded. For most measures, we calculated summary metrics for all observations quarterly (eg, participant 1 as of April 8, 2016; July 7, 2016; and October 5, 2016).

The goal of this work was not to identify explanatory variables for DKA risk but to develop a high-performing predictive model that is feasible for clinical implementation. Clinical implementation of a predictive model requires a data pipeline and analytic approach that can manage the biased *missingness* that characterizes data in EHR systems (ie, some important observations are recorded infrequently and only on individuals who are sick or who access care within an observation window). We used a simple imputation approach to solve this problem. We assumed that to meet our objective, absent observations in the EHR for any data feature in any quarter could be adequately represented by an individual’s most recent value carried forward or by the population average for that feature when a particular variable had never been measured in the individual. When a youth did not have a certain laboratory result or vital sign recorded during a quarter, for example, the value for that quarter was imputed using the last recorded value carried forward. When no prior measurement for a laboratory or vital sign was available, we set the imputed value to the population average for that variable. On average, results for approximately 4.28% (58,897/1,377,000) of all available laboratory tests performed on the cohort during the total observation period were present during any given quarter (ie, 1,318,103/1,377,000, 95.72% of laboratory values were imputed per quarter). A total of 10.61% (146,053/1,377,000) of laboratory values were imputed using an earlier value and 85.12% (1,172,050/1,377,000) were imputed using the population average. For vital signs, on average, approximately 44.66% (49,197/110,160) were present during a given quarter, 15.48% (17,053/110,160) were imputed using an earlier value, and 39.86% (43,910/110,160) were imputed using the population average.

We used counts to represent certain data types. The counts per quarter for the 50 most common medications, counts of all medical visits, and counts by type of visit (daytime, ambulatory, emergency, inpatient, outpatient, and other) were recorded. We also included the number of previous DKA admissions and the number of days since the last DKA admission, which was capped at 365 days for those who did not experience DKA during the previous year. For the training, partial out-of-sample (OOS-P), and full out-of-sample (OOS-F) cohorts, the number of previous DKA admissions only included DKA admissions from quarters 1 to 7, 3 to 9, and 10 to 15, respectively.

Glycated hemoglobin (HbA_1c_) is an important biomarker that is the current gold standard for estimating average glycemic control over approximately the prior 90 days. Diagnostic and quarterly HbA_1c_ values were included as clinical features. Diagnostic HbA_1c_ was defined as the youth’s first recorded HbA1_c_ result, which in most cases reflected HbA_1c_ at the time of T1D diagnosis before the initiation of diabetes treatment. If HbA_1c_ was missing for a given quarter, the value was linearly interpolated between the closest actual observations before and after that quarter. Otherwise, the most recent HbA_1c_ from the prior 2 quarters was used. If no recent HbA_1c_ was present, we imputed missing HbA_1c_ values using the median HbA_1c_. Youth were stratified by age at encounter before imputing missing values to account for age-specific HbA_1c_ variation. On average, approximately 60.69% (8357/13,770) of quarters had an available HbA_1c_ value, 6.81% (938/13,770) of HbA_1c_ values were imputed using linear interpolation, 10.16% (1399/13,770) were imputed using the last recorded value, and 22.34% (3076/13,770) were imputed using the population average by age.

To mitigate the possibility of overfitting (strong performance in the training data but poor performance in unseen data sets) and to improve the model training process, we limited the total number of clinical features in the trained model to the most common values observed across the population. Threshold numbers were chosen to determine how many CUI codes, laboratory results, vital signs, medications, patient-reported outcomes, patient-reported outcome surveys, and features derived from NLP of free-text clinical notes would be included in model development. All values were subsequently scaled to ensure that none of them would overpower the model. The LSTM model considered >500 features per observation period. A random forest model with the same input features and outcomes was trained in parallel to allow estimation of feature importance.

### Outcome Definition

The LSTM model estimated the time to DKA-related hospitalization using the Weibull distribution, which is a continuous probability curve often used by engineers to analyze the time to failure for various machines and materials [15,16]. After determining the Weibull distribution for DKA-related hospitalization, we calculated the youth’s cumulative daily probability of DKA-related hospitalization within 180 days as our final model output.

### LSTM Model Development and Validation

**Figure 1:**
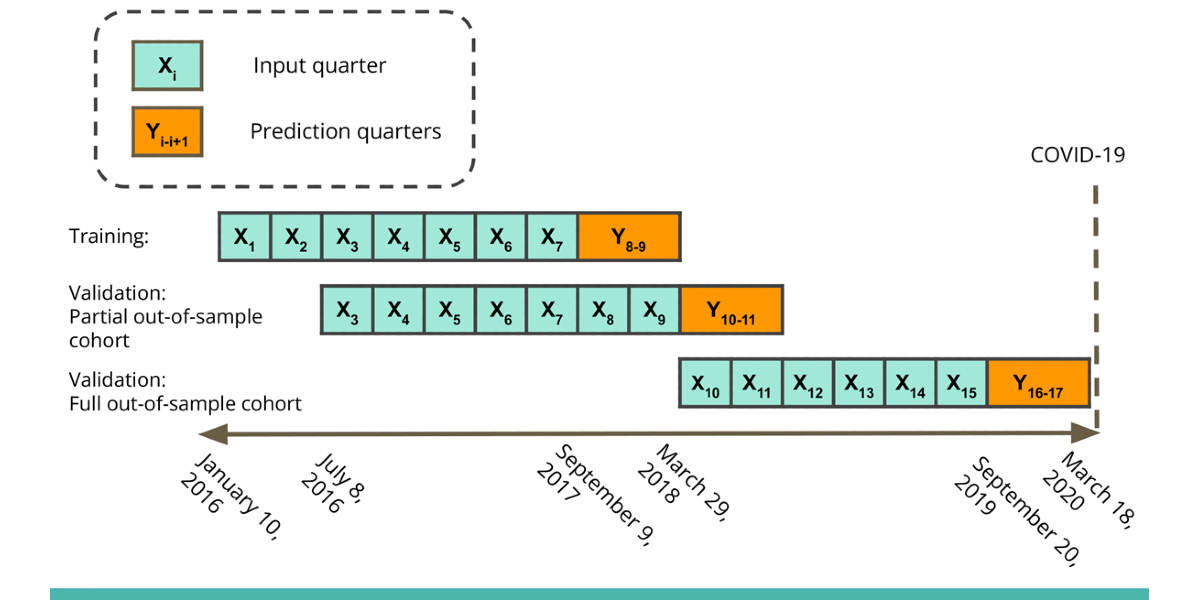
Long short-term memory structure used to predict diabetic ketoacidosis–related hospitalization within the subsequent 180 days in youth with type 1 diabetes in the training, partial out-of-sample, and full out-of-sample validation cohorts.

Figure 1 illustrates the LSTM data structure. We created a training set using 7 consecutive 90-day periods of input data from quarters 1 to 7 (January 10, 2016, to September 30, 2017) for 1377 youths, and we predicted the risk of DKA-related hospitalization in the next 180 days (quarters 8 and 9). We first sought to evaluate whether an OOS-P cohort could be used to validate the model performance using input data from quarters 3 to 9 (July 8, 2016, to March 29, 2018) for 1505 youths. Our rationale for creating an OOS-P cohort was that this approach might be the ideal one for monitoring the model performance in an ongoing way during clinical implementation. Moreover, many clinics aiming to adopt this model may have limited data available for fine-tuning, validating, and monitoring the approach. The OOS-P cohort included the original training cohort (n=1377); an additional 72 new, model-naive youths who were randomly withheld from the training cohort (72/1377, 5.23% of the total); and 56 model-naive youths who entered the cohort as those with new T1D diagnoses during quarters 8 or 9. We used the OOS-P validation data set to assess risk in a new 180-day observation window: quarters 10 and 11 (March 3, 2018, to September 25, 2018).

We then performed a gold standard OOS-F validation of the model using data from quarters 10 to 15 (March 29, 2018, to September 20, 2019) for 354 new, model-naive individuals. The OOS-F cohort included 114 model-naive youths who were excluded from the training cohort, either because they were part of the planned, randomly selected 5.23% (72/1377) who were excluded or because they were not yet eligible for inclusion in model training because of the T1D diagnosis date occurring between quarters 1 and 7; an additional 240 youths entered the cohort (as those with new diagnoses or transfers of care) in quarters 10 to 15. We used the OOS-F validation data set to assess risk in quarters 16 to 17 (September 29, 2019, to March 18, 2020).

### Statistical Analysis

Descriptive statistics were expressed as frequencies, percentages, medians, and IQRs. We created lists for the top 5.32% (80/1505) and 5.1% (18/354) of youth in each validation cohort with the highest cumulative probability of DKA-related hospitalization. As a result, list sizes of 80 and 18 were determined to be the most accurate approximations for the number of individuals who experienced DKA-related hospitalization within 180 days in the OOS-P and OOS-F validation cohorts, respectively. We compared youth within and outside the top 80 and top 18 for the OOS-P and OOS-F validation cohorts, respectively, using chi-square, Fisher exact, or 2-sample Wilcoxon rank-sum (Mann-Whitney) tests. To evaluate the proposed LSTM model’s efficiency, we calculated precision (positive predictive value) and recall (sensitivity). We calculated precision as the proportion of members within a segment of the rank-ordered list (members 1 to 5, 1 to 10, etc) who experienced DKA-related hospitalization. We calculated recall by counting the number of youths in the rank-ordered list who actually experienced DKA-related hospitalization and then dividing by the list size (80 or 18, the number of admissions per 180-day period) for the OOS-P and the OOS-F validation cohorts, respectively. We produced the area under the receiver operating characteristic (AUROC) curve and the area under the precision-recall curve (AUPRC) to display the diagnostic performance of the machine learning model. All summary statistics and analyses were conducted using Stata/SE software (version 15.1; StataCorp LLC). The *P* values ≤.05 were considered statistically significant.

### Ethics Approval

Clinical and model output data were coded and collected in an institutional review board–approved research data repository (IRB #11120355) that met the requirements for a waiver of written informed consent as outlined in US Department of Health and Human Services regulation 45 CFR 46.116.

## Results

### Overview

Table 1 shows the demographics and characteristics of the training, OOS-P validation, and OOS-F validation cohorts. For the OOS-P and OOS-F validation cohorts, the rate of DKA-related hospitalizations within the 180-day observation period was 5% (quarters 10 and 11) and 5% (quarters 16 and 17); the median age was 13.7 (IQR 11.3-15.8) years and 13.1 (IQR 10.7-15.5) years; 48.77% (734/1505) and 45.8% (162/354) were female, 80.26% (1208/1505) and 75.1% (266/354) were White; 50.56% (761/1505) and 50.3% (178/354) had private insurance; the median duration of T1D was 4.8 (IQR 2.5-7.9) years and 0.9 (IQR 0.6-1.6) years; 58.2% (876/1505) and 22.9% (79/344) were on an insulin pump; 29.1% (438/1505) and 40.9% (145/354) were documented as using a continuous glucose monitoring (CGM) device; median HbA_1c_ levels at enrollment were 8.6% (IQR 7.6%-9.8%) and 8.1% (IQR 6.9%-9.5%); and 14.15% (213/1505) and 12.7% (45/354) had a prior (after the T1D diagnosis) DKA-related hospitalization, respectively.

**Table 1:** Demographics and characteristics of the long short-term memory model.

			Training cohort (n=1377)	Partial out-of-sample validation cohort (n=1505)	Full out-of-sample validation cohort (n=354)	*P* value^a^	
Sex (female), n (%)			670 (48.66)	734 (48.77)	162 (45.76)	.31	
Age (years), median (IQR)			13.3 (10.9-15.4)	13.7 (11.3-15.8)	13.1 (10.7-15.5)	.009	
**Ethnicity** **, n (%)**							.03
	Non-Hispanic		1278 (92.81)	1399 (92.96)	317 (89.55)		
	Hispanic		99 (7.19)	106 (7.04)	37 (10.45)		
**Race, n (%)**							.04
	Asian		10 (0.73)	10 (0.66)	4 (1.13)		
	Black or African American		120 (8.71)	132 (8.77)	28 (7.91)		
	White		1104 (80.17)	1208 (80.27)	266 (75.14)		
	Other race		15 (1.09)	16 (1.06)	5 (1.41)		
	Unknown		128 (9.3)	139 (9.24)	51 (14.41)		
**Insurance type, n (%)**							.001
	Public		676 (49.09)	742 (49.30)	169 (47.74)		
	Private		699 (50.76)	761 (50.56)	178 (50.28)		
	Self-pay		2 (0.15)	2 (0.13)	7 (1.98)		
**Medical records**							
	Chronic conditions^b^, n (%)		906 (65.8)	1010 (67.11)	167 (47.18)	<.001	
	**Number of previous DKAs^c^, n (%)**						<.001
		0	1212 (88.02)	1292 (85.85)	309 (87.29)		
		1	61 (4.43)	82 (5.45)	36 (10.17)		
		2	65 (4.72)	75 (4.98)	3 (0.85)		
		≥3	39 (2.83)	56 (3.72)	6 (1.69)		
	Youth without DKA in prior 365 days, n (%)		1268 (92.08)	1384 (91.96)	325 (91.81)	.93	
	DKA admission in subsequent 180 days, n (%)		68 (4.94)	80 (5.32)	18 (5.08)	.86	
	**Last glycated hemoglobin (%) measured^d^, median (IQR)**		8.6 (7.6-9.7)	8.6 (7.6-9.8)	8.1 (6.9-9.5)	<.001	
		International Federation of Clinical Chemistry (mmol/mol)	70 (60-83)	70 (60-84)	65 (52-80)		
	Days since last glycated hemoglobin measured^d^, median (IQR)		54 (29.5-86)	58 (30-98)	63 (30-95)	.51	
	Age at T1D^e^ diagnosis in years, median (IQR)		8.2 (5.4-10.8)	8.4 (5.6-11.0)	11.1 (9.0-13.9)	<.001	
	Duration of T1D in years, median (IQR)		4.6 (2.3-7.6)	4.8 (2.5-7.9)	0.9 (0.6-1.6)	<.001	
**Insulin delivery method^f^, n (%)**							<.001
	MDI^g^		608 (44.25)	625 (41.56)	265 (77.03)		
	Insulin pump		761 (55.39)	876 (58.24)	79 (22.97)		
	No insulin		5 (0.36)	3 (0.2)	0 (0)		
**Glucose monitoring method^h^, n (%)**							<.001
	CGM^i^		350 (25.42)	438 (29.10)	145 (40.96)		
	SMBG^j^		1027 (74.58)	1067 (70.9)	209 (59.04)		

^a^*P* values were generated via chi-square, Fisher exact, or 2-sample Wilcoxon rank-sum (Mann-Whitney) tests comparing partial and full out-of-sample validation cohorts.

^b^Chronic conditions were documented if any International Classification of Diseases codes were in the chronic condition indicator or warehouse, excluding diabetes.

^c^DKA: diabetic ketoacidosis.

^d^For the last glycated hemoglobin measurement and days since the last glycated hemoglobin measurement: training cohort, n=1336; partial out-of-sample validation cohort, n=1490; and full out-of-sample validation cohort, n=347.

^e^T1D: type 1 diabetes.

^f^For the insulin delivery method: training cohort, n=1374; partial out-of-sample validation cohort, n=1504; and full out-of-sample validation cohort, n=344.

^g^MDI: multiple daily injections.

^h^For continuous glucose monitoring method: training cohort, Dexcom (G4, G5 or G6): n=239 and Medtronic (Guardian): n=111; partial out-of-sample validation cohort, Dexcom (G4, G5 or G6): n=321 and Medtronic (Guardian): n=117; and full out-of-sample validation cohort, Dexcom (G4, G5 or G6): n=117, Medtronic (Guardian): n=13, and Freestyle Libre: n=15.

^i^CGM: continuous glucose monitoring.

^j^SMBG: self-monitoring of blood glucose.

### Precision, Recall, and AUCs

To measure the performance of the LSTM model, we calculated precision and recall across various segments of the rank-ordered lists (Tables 2 and 3). As DKA-related hospitalization occurred in approximately 5.62% (98/1745) of youth in our study, we generated rank-ordered lists of the top 5.32% (80/1505) and 5.1% (18/354) of youth in the OOS-P (n=80) and OOS-F (n=18) validation cohorts, respectively, with the highest cumulative probability of DKA-related hospitalization. Those labeled with the highest probability of DKA-related hospitalization were assigned the highest ranks in each list. In the OOS-P validation cohort, for the list segment representing positions 1 to 10, precision was 100%, indicating that all 10 list members experienced DKA-related hospitalization. Recall for the same segment was 13% because the 10 members represented 10 (13%) of the 80 members of the total population who experienced DKA-related hospitalization in the subsequent 180 days. For list segments 1 to 25 and 1 to 80, precision was 56% and 33%, whereas recall was 18% and 33%, respectively. In the OOS-F validation cohort, for the list segment representing positions 1 to 5, precision was 80%, indicating that 4 of the 5 list members experienced DKA-related hospitalization. Recall for the same segment was 22% because the 4 members represented of the 18 members of the total population who experienced DKA-related hospitalization in the subsequent 180 days. For list positions 1 to 10 and 1 to 18, precision was 60% and 50%, whereas recall was 33% and 50%, respectively.

**Figure 2:**
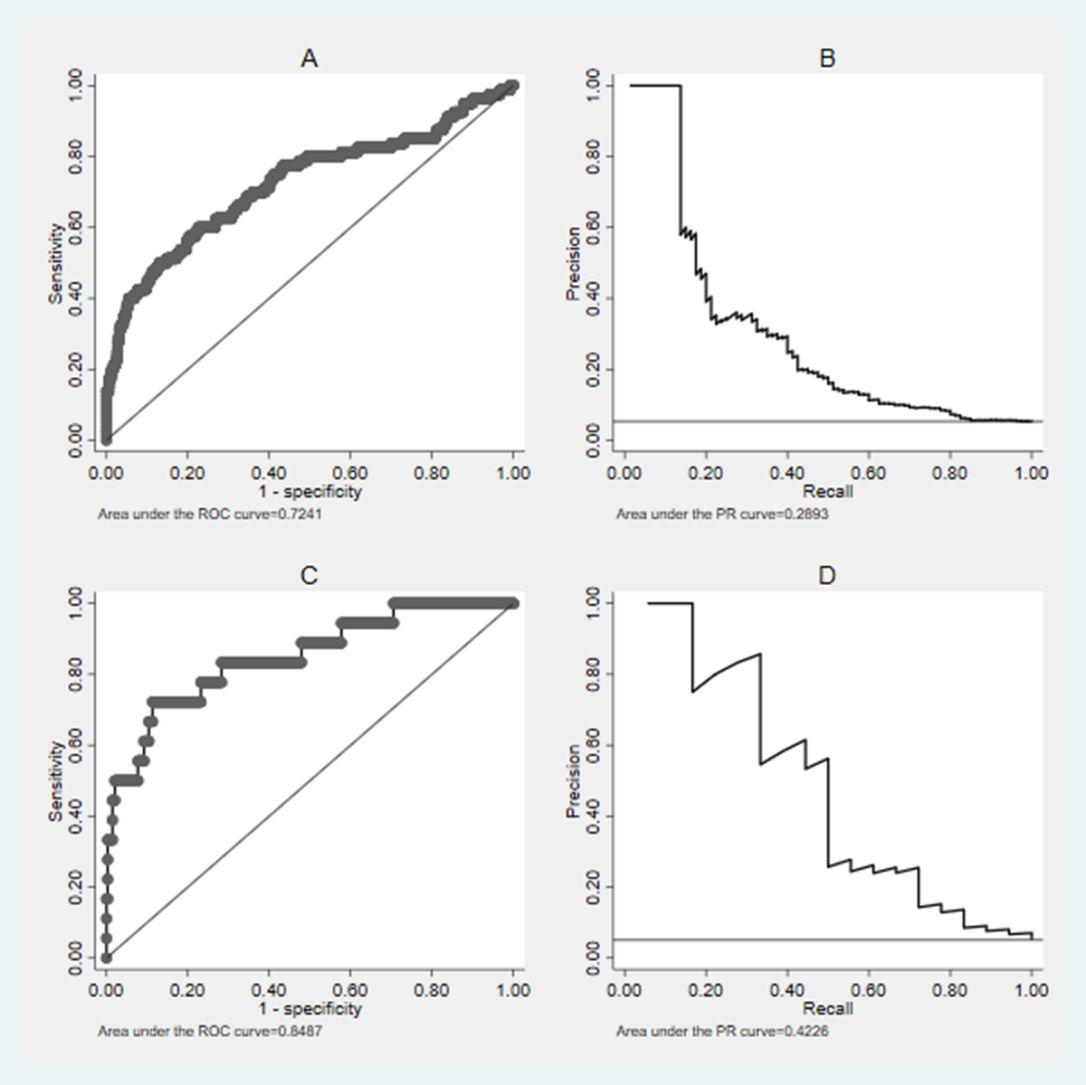
Area under the receiver operating characteristic (AUROC) curve and area under the precision-recall (PR) curve for the prediction of diabetic ketoacidosis–related hospitalization within the subsequent 180 days in youth with type 1 diabetes. For the partial out-of-sample validation cohort: (A) area under the ROC curve=0.72 and (B) area under the PR curve=0.29. For the full out-of-sample validation cohort: (C) area under the ROC curve=0.85 and (D) area under the PR curve=0.42.

Next, we generated a receiver operating characteristic curve to examine the relationship between sensitivity and specificity at various cutoff values. Owing to data imbalance, we also generated a precision-recall curve to examine the relationship between the true positive rate (recall) and the positive predictive value (precision) at different probability thresholds. The model demonstrated an AUROC of 0.72 and 0.85 and an AUPRC of 0.29 and 0.42 for the OOS-P and OOS-F validation cohorts, respectively (Figure 2).

**Table 2:** Precision and recall according to diabetic ketoacidosis (DKA) admission frequency in the long short-term memory (LSTM) model for the partial out-of-sample validation cohort.

List size	Members with subsequent DKA within 180 days, n	Precision (actual members/list size), n (%)	Recall (actual members/80), n (%)
5	5	5 (100)	5 (6)
10	10	10 (100)	10 (13)
25	14	14 (56)	14 (18)
50	17	17 (34)	17 (21)
80	26	26 (33)	26 (33)

**Table 3:** Precision and recall according to diabetic ketoacidosis (DKA) admission frequency in the long short-term memory (LSTM) model for the full out-of-sample validation cohort.

List size	Members with subsequent DKA within 180 days, n	Precision (actual members/list size), n (%)	Recall (actual members/18), n (%)
5	4	4 (80)	4 (22)
10	6	6 (60)	6 (33)
18	9	9 (50)	9 (50)

Next, we compared youth within the top 5% with youth outside the top 5% for the OOS-P (youth within the top 80 vs outside the top 80) and OOS-F (youth within the top 18 vs outside the top 18) validation cohorts (Table 4). For the OOS-P validation cohort, we observed significant differences in sex, age in years, race, insurance, proportion with other chronic conditions, proportion with any previous DKA episodes, proportion with no DKA episodes in the previous 365 days, last HbA_1c_, duration of T1D in years, and proportion using an insulin pump or a CGM device between those in the top 5% and those not in the top 5% by risk of DKA-related hospitalization. We also observed a difference in the proportion of youth with DKA-related hospitalization in the subsequent 180 days. In the OOS-F validation cohort, we observed differences in sex, race, proportion with other chronic conditions, proportion with any previous DKAs, proportion with no DKA episodes in the previous 365 days, last HbA_1c_, and proportion using a CGM device. We also observed differences in the proportion of youth with DKA-related hospitalization in the subsequent 180 days.

**Table 4:** Comparison of youth outside to within top 80 and top 18 ranks, respectively, for the partial and full out-of-sample validation cohorts.

			Partial out-of-sample validation cohort (n=1505)					Full out-of-sample validation cohort (n=354)			
			Outside top 80 (n=1425)	Top 80 (n=80)	*P* value^a^		Outside top 18 (n=336)		Top 18 (n=18)	*P* value^a^	
Sex (female), n (%)			677 (47.51)	57 (71.25)	<.001		149 (44.35)		13 (72.22)	.03	
Age (years), median (IQR)			13.6 (11.3-15.7)	15.3 (13.5-16.6)	<.001		12.9 (10.6-15.4)		14.4 (12.7-16.1)	.08	
**Ethnicity, n (%)**						.65					.42
	Non-Hispanic		1323 (92.84)	76 (95)			302 (89.88)		15 (83.33)		
	Hispanic		102 (7.16)	4 (5)			34 (10.12)		3 (16.67)		
**Race, n (%)**						<.001					.03
	Asian		10 (0.7)	0 (0)			4 (119)		0 (0)		
	Black or African American		102 (7.16)	30 (37.5)			24 (7.14)		4 (22.22)		
	White		1169 (82.04)	39 (48.75)			257 (76.49)		9 (50)		
	Other race		16 (1.12)	0 (0)			4 (1.19)		1 (5.56)		
	Unknown		128 (8.98)	11 (13.75)			47 (13.99)		4 (22.22)		
**Insurance type, n (%)**						<.001					.09
	Public		672 (47.16)	70 (87.5)			157 (46.73)		12 (66.67)		
	Private		751 (52.7)	10 (12.5)			173 (51.49)		5 (27.78)		
	Self-pay		2 (0.14)	0 (0)			6 (1.79)		1 (5.56)		
**Medical records**											
	Chronic conditions^b^, n (%)		938 (65.82)	72 (90)	<.001		151 (44.94)		16 (88.89)	<.001	
	**Number of previous DKAs^c^, n (%)**					<.001					<.001
		0	1281 (89.89)	11 (13.75)			301 (89.58)		8 (44.44)		
		1	69 (4.84)	13 (16.25)			32 (9.52)		4 (22.22)		
		2	53 (3.72)	22 (27.50)			1 (0.3)		2 (11.11)		
		≥3	22 (1.54)	34 (42.5)			2 (0.6)		4 (22.22)		
	Youth without DKA in prior 365 days, n (%)		1361 (95.51)	23 (28.75)	<.001		317 (94.35)		8 (44.44)	<.001	
	DKA admission in subsequent 180 days, n (%)		54 (3.79)	26 (32.5)	<.001		9 (2.68)		9 (50)	<.001	
	**Last glycated hemoglobin (%) measured^d^, median (IQR)**		8.5 (7.5-9.6)	11.5 (10.8-13)	<.001		8.0 (6.9-9.4)		10.6 (8.8-11.9)	<.001	
		International Federation of Clinical Chemistry (mmol/mol)	69 (58-81)	102 (95-119)			64 (52-79)		92 (73-107)		
	Days since last glycated hemoglobin measured^d^, median (IQR)		59 (30-98)	51.5 (28.5-101)	.42		64 (30-95)		31 (23-81)	.12	
	Age at T1D^e^ diagnosis in years, median (IQR)		8.3 (5.6-10.9)	9.4 (6.5-11.7)	.07		11.1 (9.0-13.8)		12.5 (9.6-14.3)	.33	
	Duration of T1D in years, median (IQR)		4.8 (2.3-7.9)	5.2 (3.7-7.4)	.04		0.9 (0.6-1.5)		1.2 (0.4-2.5)	.79	
**Insulin delivery method^f^, n (%)**						.02					.13
	MDI^g^		580 (40.73)	45 (56.25)			250 (76.22)		15 (93.75)		
	Insulin pump		841 (59.06)	35 (43.75)			78 (23.78)		1 (6.25)		
	No insulin		3 (0.21)	0 (0)			0 (0)		0 (0)		
**Glucose monitoring method^h^, n (%)**						<.001					.05
	CGM^i^		433 (30.39)	5 (6.25)			142 (42.26)		3 (16.67)		
	SMBG^j^		992 (69.61)	75 (93.75)			194 (57.74)		15 (83.33)		

^a^*P* values were generated using chi-square, Fisher exact, or 2-sample Wilcoxon rank-sum (Mann-Whitney) tests comparing outside top 80 and top 80 groups for the partial out-of-sample validation cohort and outside top 18 and top 18 groups for the full out-of-sample validation cohort.

^b^Chronic conditions were documented if any International Classification of Diseases codes were in the chronic condition indicator or warehouse, excluding diabetes.

^c^DKA: diabetic ketoacidosis.

^d^For the last glycated hemoglobin measured and days since the last glycated hemoglobin measurement: partial out-of-sample validation cohort (outside top-80), n=1410 and full out-of-sample cohort (outside top-18), n=329.

^e^T1D: type 1 diabetes.

^f^For insulin delivery method: partial out-of-sample validation cohort (outside top 80), n=1424; full out-of-sample validation cohort (outside top 18), n=328; and full out-of-sample validation cohort (top 18), n=16.

^g^MDI: multiple daily injections.

^h^For continuous glucose monitoring method: partial out-of-sample validation cohort (outside top 80), Dexcom (G4, G5 or G6): n=317 and Medtronic (Guardian): n=116; partial out-of-sample validation cohort (top 80), Dexcom (G4, G5 or G6): n=4 and Medtronic (Guardian): n=1; full out-of-sample validation cohort (outside top 18), Dexcom (G4, G5 or G6): n=116, Medtronic (Guardian): n=13, and Freestyle Libre: n=13; and full out-of-sample validation cohort (top 18), Dexcom (G4, G5 or G6): n=1, Medtronic (Guardian): n=0, and Freestyle Libre: n=2.

^i^CGM: continuous glucose monitoring.

^j^SMBG: self-monitoring of blood glucose.

### Feature Weights

To determine the features that most impacted the predictions, we applied a random forest model using the same input features that were used in the LSTM model. The 10 top-weighted features were diagnostic HbA_1c_, HbA_1c_ in the past year, HbA_1c_ from the last 90 days, age at prediction, heart rate, number of previous DKA admissions, days since DKA, BMI, Immunoglobulin A test in the past year, and median household income (additional data are provided in Multimedia Appendix 1).

## Discussion

### Principal Findings

We developed and examined the initial validity of a deep learning model to predict hospitalization for DKA within 180 days among youth with previously diagnosed T1D. We examined model performance using lists containing the rank-ordered top 5% of youth with the highest probability of hospitalization (selected to match the 180-day incidence of DKA among established patients aged 8 to 18 years in the clinic). AUPRC showed a steep drop in precision to achieve recall measures of approximately >10% and >33% in the OOS-P and OOS-F validation cohorts, respectively. Precision increased progressively as the threshold for inclusion rose on the rank-ordered list (including all 80, vs the top 25, vs the top 10 youth for the OOS-P cohort), suggesting the model’s ability to produce variably risk-enriched cohorts of individuals who might be considered eligible for more intensive intervention. Compared with the incidence of DKA-related hospitalization in this study of 0.05, the AUPRC values of 0.29 and 0.42 in the OOS-P and OOS-F validation cohorts, respectively, are significantly larger. Receiver operating characteristic curves for the OOS-P and OOS-F validation cohorts demonstrated that the model had a 72% and 85% probability, respectively, of identifying youth with T1D who will experience DKA-related hospitalization within 180 days.

Prior multinational and single-center studies have shown that multiple demographic and clinical care factors are associated with increased risk of hospitalization for DKA in United States– and European-based populations: hospital admissions for DKA in the prior 12 months, nonprivate insurance, elevated HbA_1c_, racial and ethnic minority individuals, lower household income, mental health comorbidities, female sex, missed endocrine appointments, higher insulin doses, and insulin delivery by injection [17-21]. In a multinational registry of approximately 50,000 children, Maahs et al [22] identified female sex, ethnic minority groups, and individuals with HbA_1c_ ≥7.5% (≥58 mmol/mol) as having an increased risk of experiencing DKA. Their aim was to identify the factors associated with DKA and not to implement a model that clinically predicts future DKA admissions. They used a limited number of discrete variables available in the EHR and cross-sectional data, which did not consider changes in predictors or the recurrence of discrete events over time. In addition, most of the factors were not modifiable. Notably, our comprehensive prediction model uses a greater variety of data that are widely recorded in EHRs and identifies a considerable number of at-risk youth who did not experience DKA-related hospitalization in the previous 12 months. The rate of DKA in this cohort (5%) is consistent with the annual rates (1% to 15% per established patient per year) reported in prior studies [19,23,24]. Prior work suggests that 20% of annual admissions for DKA involve readmissions of the same individual within 1 year [25]. Youth in the top 5% of the rank-ordered lists for both validation cohorts consisted of more female individuals, were older, were more often racial and ethnic minority individuals, and more frequently experienced other chronic conditions. They also had a higher prevalence of previous DKA admissions, a higher prevalence of DKA admission in the subsequent 180 days, elevated HbA_1c_, and a lower proportion of CGM use compared with youth outside the top 5%. Compared with the OOS-P validation cohort, fewer youth in the OOS-F cohort were on insulin pumps. This is likely related to the shorter duration of T1D in the OOS-F cohort versus the training and OOS-P cohorts.

Few studies have sought to develop and validate risk prediction models for DKA that could be deployed in clinical care. One study developed a multivariable prediction model using generalized estimating equations to predict DKA events within the next 12 months among youth with T1D. In that study, hospital admission in the prior year, HbA_1c_, nonprivate insurance, female sex, and racial and ethnic minority individuals predicted DKA admissions, whereas age, duration of diabetes, and number of office visits in the prior year did not. The AUROC curve for that model was 0.735 to 0.746, compared with 0.72 to 0.85 for the model used in this study. The prior model resulted in a 5-fold risk-enriched population, which is comparable with our model’s overall performance in the 5.62% (98/1745) of individuals with the highest risk probabilities. In contrast, the approach used in this study allows significantly greater risk enrichment (16 to 20 fold) if one focuses on patients with higher ranks on the rank-ordered list by probability of admission [26]. An independent study of youth reported on the development of a risk index that achieved an AUROC curve of 0.709. However, the generation of that index required the use of a 20- to 30-minute psychosocial screening tool, which could be a significant barrier to clinical adoption [27]. Another study reported the performance of different machine learning approaches in predicting DKA among adults with T1D using EHR data and a small set of hand-selected features [28]. This nested case-cohort study leveraged the Optum database of EHR records, which consisted of 3400 potential DKA cases and 11,780 control cases. The authors found that different machine learning techniques demonstrated similar performance and identified overlapping but different top 10 predictors. As their purpose was to identify factors associated with hospital admission for DKA, they did not report a prespecified observation window for predicting the outcome. This omission may make the models, as reported, challenging to translate into practice by clinicians who want to forecast the probability of hospital admission for DKA within defined periods.

This study differs substantially from prior studies in its focus on predicting DKA events within 180 days in a pediatric population; in its model development approach, which combined discrete data elements with features derived from NLP of free-text clinical documents; in the diversity and scale of data features used to create the model; and in the use of LSTM, which retains a memory of more distal historical events when weighting features. The approach used in this study is also novel because it introduces the use of a simple-to-interpret list that is rank ordered by the probability of hospital admission, allowing clinicians to choose the number of top-ranked patients they will select for intervention based on capacity. The threshold rank that clinicians use to select individuals for intervention is directly tied to the level of risk enrichment (eg, 5.5 to 20 fold) they will achieve in the target cohort, which makes it easier to determine the number needed to treat to have a chance of preventing 1 hospitalization for DKA. For example, using the OOS-P findings, targeting youths 1 to 10 on the rank-ordered list would require treating only 1 youth to potentially prevent DKA-related hospitalization. In contrast, one would have to treat 3 youths from individuals comprising the top 5% of risk (ranks 1 to 80 in the OOS-P cohort) to have a chance of preventing DKA-related hospitalization in at least 1 youth. How clinicians use the rank-ordered list can thus impact the cost-effectiveness of any chosen interventional strategy.

These results are clinically meaningful because they offer a practical approach for continuous DKA risk stratification in youth within a T1D clinical population. Creating rank-ordered lists of youth based on the probability of admission is clinically intuitive and adaptable to clinical workflows that involve care navigation (enrolling youth in specific care pathways based on risk or established eligibility criteria). Even clinics with limited resources can benefit from this approach by a priori defining the number of youths per 6-month period for which they have the capacity to intervene. Longitudinal DKA risk scores based on the probability of hospital admission can be tracked as a process metric to drive resource allocation and quality improvement projects. When health systems apply deep learning, best practices should be followed to protect data to uphold privacy, design transparent and interpretable models, and prevent bias or discrimination among groups. Health care providers and developers need to collaborate, be critical, and be discretionary regarding the application of artificial intelligence (AI) in scenarios where human health and well-being are impacted; they should not simply defer to AI outputs [29]. For example, predictive models generated via deep learning may include multiple variables, such as race and ethnicity and socioeconomic status, as input features that are used to improve model prediction. Predicted probabilities and model performance should be examined across segments of the population by age, sex, race and ethnicity, insurance type, or socioeconomic status to uncover potential health inequities or model bias. The identification of inequities or model bias in specific groups can drive quality improvement projects to rectify them.

Currently, clinicians have limited knowledge about how to prevent hospitalization for DKA. Harris et al [30] developed and evaluated the Novel Interventions for Children’s Healthcare program as an approach to preventing hospital admissions in youth with chronic diseases. Although the start-up cost to health systems or payers can be a barrier to adoption, this remains a promising approach [30-33]. Others have reported case studies on the successful use of remote patient monitoring in preventing DKA among adults with T1D [34]. One study demonstrated that quality improvement methods, with the implementation of longitudinal multiple care delivery interventions, can reduce the rate of DKA admissions in a clinical population of youth with T1D [9].

### Limitations and Strengths

This study must be considered in the context of its notable limitations and strengths. One limitation of this study is that the data and source population were derived from a regional clinic network located in the Midwestern United States; therefore, the findings may not be generalizable outside of this network’s catchment area. Future research should replicate this strategy in other geographic areas and health systems, including those using alternate EHR systems. Diabetes self-management device data were not included in this study; future studies should evaluate the inclusion of this information on model performance. Hundreds of variables were considered, which could lead to overfitting. Although we addressed this by performing an out-of-sample validation on model-naive individuals, future research should still examine this model’s performance in new institutional data sets. Another limitation lies in the use of either the most recent value carried forward or the population average as a means of interpolating missing data. Future studies should consider other methods to address missingness. Finally, we did not seek to develop an explainable model, which may limit clinicians’ trust. The use of more explainable AI models has been proposed to improve the trust of clinicians and other stakeholders [35]. These models may help clinicians identify characteristics that are heavily weighted in the prediction. For instance, it would be useful to determine whether CGM use contributes to the assignment of lower risk by the prediction model. Other researchers have experimented with various methods (the Shapley additive explanations algorithm) to achieve explainable LSTM models [36]. A future goal is to further validate feature sets that are heavily weighted in the prediction. These features could represent valuable targets for intervention.

The strengths of this study include the novel application of advanced machine learning to predict pediatric health outcomes and the quantity and variety of data evaluated compared with previous studies. Although clinical researchers have minimally used recurrent neural networks and LSTMs with medical data, opportunities exist to examine and highlight this approach for forecasting outcomes. For example, using a Weibull loss function could theoretically allow for the prediction of the probability of admission along with the time until likely admission [15,16]; this could enable the development and dissemination of just-in-time interventions to prevent DKA. Our simple imputation approach for handling missing data is another strength, enabling the model to predict the risk for youth with fewer measurements owing to reduced access to care. Risk indices that do not use imputation to address missing data among repeated measures may exclude susceptible youth who demonstrate reduced access to care. Another strength is the ability of advanced machine learning models such as LSTMs to process robust and diverse data sets with large numbers of variables per participant, even when some data are missing or inaccurate [13]. We also included features derived from NLP of free-text clinical documents, allowing a largely untapped source of clinical data from the EHR to be considered during predictive model development. Future studies should examine the relative importance of NLP- and non–NLP-derived features. Finally, we validated the predictive model using a model-naive out-of-sample cohort.

### Conclusions

Clinicians can leverage advanced machine learning to identify and rank individuals at the highest risk of experiencing DKA. We found that an LSTM model identified individuals at the highest risk of experiencing DKA-related hospitalization with reasonable precision. We proposed that clinics may apply the model used in this study to generate monthly rank-ordered lists by the probability of DKA-related hospitalization to identify at-risk individuals for targeted intervention. Clinics can determine the number of patients per month or quarter who can receive an intervention based on the available resources. This will enable future research that designs and tests novel interventions to prevent DKA-related hospitalization in those at risk. Future studies should refine and evaluate the performance of this LSTM model using data over a more extended period and in multiple clinics to ensure validation in racially, geographically, and socioeconomically diverse cohorts receiving care across different health systems.

## Appendix

Multimedia Appendix 1 The top 25 non–natural language processing weighted features, listed in order of descending feature importance, identified via the parallel random forest exercise.

## Abbreviations

AI: artificial intelligence
AUPRC: area under the precision-recall curve
AUROC: area under the receiver operating characteristic
CGM: continuous glucose monitoring
CUI: Concept Unique Identifier
DKA: diabetic ketoacidosis
EHR: electronic health record
HbA1c: glycated hemoglobin
LSTM: long short-term memory
NLP: natural language processing
OOS-F: full out-of-sample
OOS-P: partial out-of-sample
T1D: type 1 diabetes
