# Supplementary material for: An “All-Data-on-Hand” Deep Learning Model to Predict Hospitalization for Diabetic Ketoacidosis in Youth With Type 1 Diabetes: Development and Validation Study
Source: JMIR Diabetes. 2023 Jul 18;8:e47592. doi: 10.2196/47592 (PMC10394604; doi:10.2196/47592)
Supplement: Multimedia Appendix 1 [file diabetes_v8i1e47592_app1.pdf]

rank	importance	feature
1	0.01712399	diagnostic_a1c
2	0.015879419	Hemoglobin_A1c
3	0.014520441	filled_a1c
4	0.014366668	age
5	0.014173298	Heart_Rate
6	0.01132428	dkas
7	0.010287862	days_since_dka
8	0.01010495	Body_Mass_Index
9	0.009441768	IgA_Historical
10	0.009338321	median_income
11	0.00923486	TSH
12	0.008886451	n_a1cs
13	0.008667697	Systolic_Blood_Pressure
14	0.00851047	VLDL
15	0.007266465	Current_Weight
16	0.006752014	FIO2
17	0.006740262	Transglutaminase_IgA
18	0.006432267	Cholesterol_Total
19	0.006300442	Diastolic_Blood_Pressure
20	0.006023742	LDL
21	0.005813155	HDL_Cholesterol
22	0.005724832	Glucose
23	0.005360407	Calcium
24	0.004900293	Creatinine_Urine_Random
25	0.004775099	Triglycerides

Note: Natural language processing derived unigrams and bigrams (one-word and two-word phrases) are excluded from this list as they are not categorizable and are only machine readable.
